# Recommendations for High-resolution Peripheral Quantitative Computed Tomography Assessment of Bone Density, Microarchitecture, and Strength in Pediatric Populations

**DOI:** 10.1007/s11914-023-00811-9

**Published:** 2023-07-10

**Authors:** L. Gabel, K. Kent, S. Hosseinitabatabaei, A. J. Burghardt, M. B. Leonard, F. Rauch, B. M. Willie

**Affiliations:** 1 Human Performance Laboratory, Faculty of Kinesiology, University of Calgary, 2500 University Dr NW, Calgary, AB T2N 1N4 Canada; 2 McCaig Institute for Bone and Joint Health and Alberta Children’s Hospital Research Institute, University of Calgary, Calgary, AB Canada; 3 Department of Pediatrics, Stanford School of Medicine, Stanford, CA USA; 4 Research Centre, Shriners Hospital for Children-Canada, Montreal, Canada; 5 Department of Biomedical Engineering, McGill University, Montreal, Canada; 6 Department of Radiology and Biomedical Imaging, University of California San Francisco, San Francisco, CA USA; 7 Department of Pediatrics, McGill University, Montreal, Canada; 8 Faculty of Dental Medicine and Oral Health Sciences, McGill University, Montreal, Canada

**Keywords:** Bone, HR-pQCT, Pediatric, Imaging

## Abstract

**Purpose of Review:**

The purpose of this review is to summarize current approaches and provide recommendations for imaging bone in pediatric populations using high-resolution peripheral quantitative computed tomography (HR-pQCT).

**Recent Findings:**

Imaging the growing skeleton is challenging and HR-pQCT protocols are not standardized across centers. Adopting a single-imaging protocol for all studies is unrealistic; thus, we present three established protocols for HR-pQCT imaging in children and adolescents and share advantages and disadvantages of each. Limiting protocol variation will enhance the uniformity of results and increase our ability to compare study results between different research groups. We outline special cases along with tips and tricks for acquiring and processing scans to minimize motion artifacts and account for growing bone.

**Summary:**

The recommendations in this review are intended to help researchers perform HR-pQCT imaging in pediatric populations and extend our collective knowledge of bone structure, architecture, and strength during the growing years.

## Introduction

Over the last decade, a surge in pediatric bone research studies using high-resolution peripheral quantitative computed tomography (HR-pQCT) has improved our understanding of how bone is accrued during childhood and adolescence. Studies highlight changes that occur with growth and maturation, including substantial gains in bone geometry, density, and strength, as well as increases in trabecular and cortical thickness and decreases in cortical porosity at the distal tibia and radius [1–4]. In pediatric clinical populations, such as children with type 1 diabetes, altered bone microarchitecture may help explain increased fracture risk [5, 6]. As imaging devices such as HR-pQCT become more commonly used, we are acquiring a better understanding of the hierarchical structure of bone [7] and the subtle adaptations in bone structure and microarchitecture that underpin changes in bone strength across the lifespan. Pediatric HR-pQCT imaging protocols are not standardized; thus, our aim is to review current pediatric approaches and provide guidance for those embarking on imaging using HR-pQCT during the growing years.

### Overview of HR-pQCT

HR-pQCT is predominantly a research tool that assesses bone density, structure, and microarchitecture of the appendicular skeleton, with a minimal effective dose and a relatively short scan time. HR-pQCT is an attractive imaging modality due to its low radiation dose and improved resolution compared with clinical quantitative computed tomography (QCT) and peripheral QCT (pQCT). The first-generation HR-pQCT (XtremeCT, Scanco Medical AG, Brütisellen, Switzerland) has an isotropic voxel size of 82 μm, minimum effective radiation dose of 3 to 5 μSv per scan, and scan time of 2.8 min per scan [8•]. Normative data for first-generation HR-pQCT distal bone outcomes are available in adolescents and young adults (9 to 21 years) [9] and adults [10]. The second-generation HR-pQCT (XtremeCTII, Scanco Medical AG, Brütisellen, Switzerland) has an enhanced isotropic voxel size of 61 μm, similar minimum effective radiation dose of 5 μSv per scan, and shorter scan time of 2.0 min per scan [8•]. The enhanced resolution of the second-generation HR-pQCT permits direct assessment of trabecular microarchitectural outcomes, in contrast to the indirect method used in the first-generation HR-pQCT that derives trabecular thickness from trabecular number and bone volume fraction [11, 12]. Cortical porosity and thickness can be quantified using both scanner generations with an automated segmentation approach [13, 14], while compressive bone strength is estimated using finite element analysis [15]. Normative data for second-generation HR-pQCT are not currently available for children or adolescents but are for distal and proximal bone outcomes in adults [16, 17]. HR-pQCT has predominantly been used to examine the distal radius or tibia, though with the longer gantry of the second-generation device, diaphyseal regions can also be examined. Despite over a decade of research using HR-pQCT in pediatric populations, comparing results between studies is challenging since acquisition and analysis protocols are not yet standardized. A major challenge for pediatric HR-pQCT imaging is bone growth, especially in longitudinal settings.

### Bone Growth During Childhood and Adolescence

**Fig. 1:**
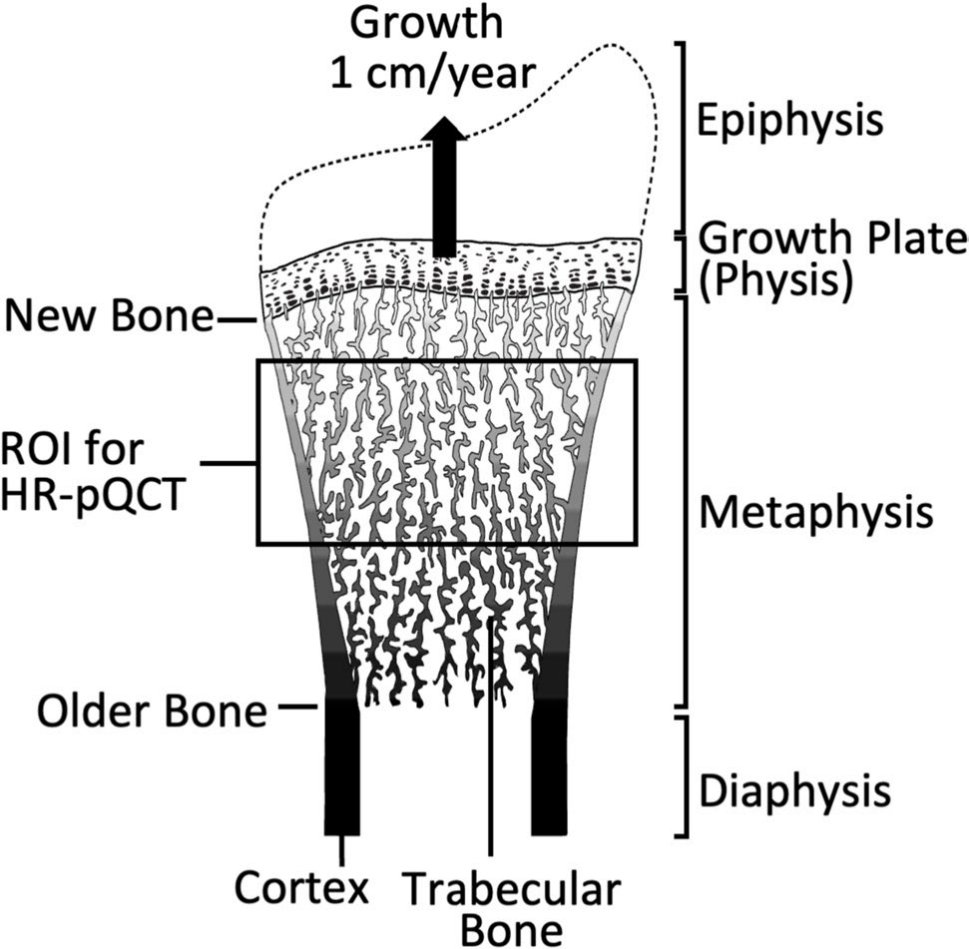
Longitudinal growth of the distal radius. The growth plate is adding cartilage tissue at a rate of about 1 cm per year. At the border between the growth plate and the metaphysis, this cartilage is replaced by bone tissue (labeled as new bone). With increasing distance from the growth plate, the bone is increasingly older (as indicated by darker color in this schematic), cortical thickness is increasing, and the outer size of the metaphysis is decreasing. The region of interest (ROI) for HR-pQCT includes metaphyseal bone that typically has been formed 1 to 2 years before, depending on how the measurement site is selected by the investigator. Modified with permission from [18]

In growing children, the distal sites are metaphyseal sites that are proximal to active growth plates. The endochondral ossification process occurring at these growth plates generates the bone tissue that is analyzed by HR-pQCT and needs to be considered when acquiring and interpreting scans [18]. Within growth plates, chondrocytes continuously proliferate, hypertrophy, and secrete extracellular matrix, while cartilage tissue is simultaneously resorbed at the border between growth plate and metaphysis (Fig. 1). Osteoblasts deposit bone matrix on top of the remaining cartilage scaffold, leading to primary trabecular bone. Primary bone quickly undergoes cycles of bone resorption and bone formation, which removes all cartilage remnants from the tissue and leads to secondary trabecular bone. Metaphyseal bone subsequently undergoes further changes, as trabeculae at the periphery of the bone coalesce, creating a metaphyseal cortex that thickens with increasing distance from the growth plate [18]. As metaphyseal bone is shaped into diaphyseal bone, trabeculae in the central part of the metaphysis undergo remodeling and are eventually removed. The overall effect of this coordinated sequence of events is to lengthen the diaphysis.

One consequence of endochondral ossification is that metaphyseal bone closer to the growth plate is “younger” than bone tissue located further away from the growth plate (Fig. 1). The age of metaphyseal bone at a given distance to the growth plate depends on the speed at which the growth plate adds new bone, rather than the chronological age of the child. For example, the distal radius growth plate adds approximately 1 cm per year to the length of the bone in a growing child [19]. Therefore, bone located 1 cm proximal to that growth plate was created 1 year ago, whereas bone 2 cm from the growth plate is 2 years old. Since HR-pQCT scan volume is approximately 1 cm, there may be no overlap between bone measured at baseline and bone measured at follow-up in longitudinal investigations (depending on the measurement time interval and maturity of the child or adolescent). Metaphyseal bone only starts to grow older in parallel with chronological age when growth plate activity stops. This is a key consideration when interpreting HR-pQCT data in pediatric studies, as only recently formed bone is analyzed while children are growing. A consequence of imaging relatively new metaphyseal bone is that the distal radius cortex remains very thin throughout the growing years and only thickens when growth ceases [18], which can make differentiating between thin cortical bone and thick trabeculae challenging. Metaphyseal development of the distal tibia mirrors that of the radius. However, whereas the distal growth plate of the radius contributes about 90% to the overall length of that bone, the distal tibia growth plate is only responsible for 20 to 50% of tibia growth, varying with the age of the child [20, 21]. Although the relative contribution of the distal and proximal growth plates to overall growth varies with age [20, 21], the overall speed of longitudinal bone growth is slower at the distal tibia than at the distal radius.

In the following sections, we outline considerations for HR-pQCT scanning in pediatric populations, including common approaches for imaging growing bone using HR-pQCT, the strengths and limitations of each approach, special cases, tips and tricks for acquiring scans, and future research directions. We hope this review provides guidance for investigators embarking on HR-pQCT scanning in pediatric populations and a springboard for continued discussions around harmonizing scanning acquisition and analysis protocols. We provide considerations for first- and second-generation HR-pQCT and refer to the devices as XCTI and XCTII, respectively.

## Overview of Current HR-pQCT Techniques

### Specifications of HR-pQCT Scanners

Technical parameters of HR-pQCT are provided in detail elsewhere [8•, 11]. In brief, HR-pQCT uses an X-ray source and detector array that rotate around the lower leg or forearm. For XCTI, the X-ray tube spans a 12.6 cm field of view (diameter of the scanner opening), maximum object length of 15.0 cm, and acquires 110 parallel CT slices stacked to form a 3D image with an isotropic voxel size of 82 μm. In XCTII, the field of view is 14.0 cm, maximum object length of 20.0 cm (XCTIIa) or 22.0 cm (XCTIIb), and the system acquires 168 parallel CT slices with an isotropic voxel size of 61 μm. The manufacturer’s standard settings, including effective energy, X-ray tube current, and integration time, also vary between scanner models. One clear advantage to the second generation XCTII is that its increased resolution (61 μm voxel size, 92.5 to 112.6 μm spatial resolution) permits direct assessment of trabecular microarchitecture, such as trabecular thickness [11, 22], whereas trabecular measures from XCTI (82 μm voxel size, 134.6 to 154.4 μm spatial resolution) were indirectly derived from bone mineral density and trabecular number [11, 22, 23]. Increased spatial resolution is particularly pertinent for assessing growing bone, as trabeculae are thinner in children, thicken during growth, and approximate the spatial resolution of XCTII (87–193 μm from age 2 to 23 years) [24]. Greater partial volume effects are also expected for children since the scanner resolution is similar to the thickness of trabeculae.

Aside from the growth plate, no highly radiation sensitive tissues exist at the tibia or radius metaphysis or diaphysis. The effective dose equivalent weighting factor for HR-pQCT yields low radiation dose values (< 3 to 5 μSv per scan). HR-pQCT radiation doses are similar to a total body scan using dual X-ray absorptiometry (DXA), less than a femoral neck DXA scan (~ 8 μSv) [25], and substantially less than a chest x-ray (~ 130 μSv) [26]. For comparison, annual radiation exposure from background radiation varies around the world, but is approximately 2400 μSv per year [27]. Scatter radiation from HR-pQCT is < 1 μSv [28].

### Image Acquisition

The main challenge facing pediatric bone research is determining where to image growing bone. The manufacturer’s standard protocol uses a fixed distance region of interest (ROI) at the distal tibia and radius, which is not optimal when scanning individuals with different limb lengths. Thus, adult protocols have moved away from using fixed distance ROIs and now recommend using a relative distance ROI from a reference point [8•]. In mature bone, the fixed or relative distance ROI captures the same volume of bone over time. However, in growing bone the fixed or relative distance ROI is a “moving target,” one that becomes more distal as the participant grows. As forearm length increases by 1 cm/year on average during adolescent growth [19], the same fixed scan location may result in markedly different contributions of cortical and trabecular compartments over time [29].

**Fig. 2:**
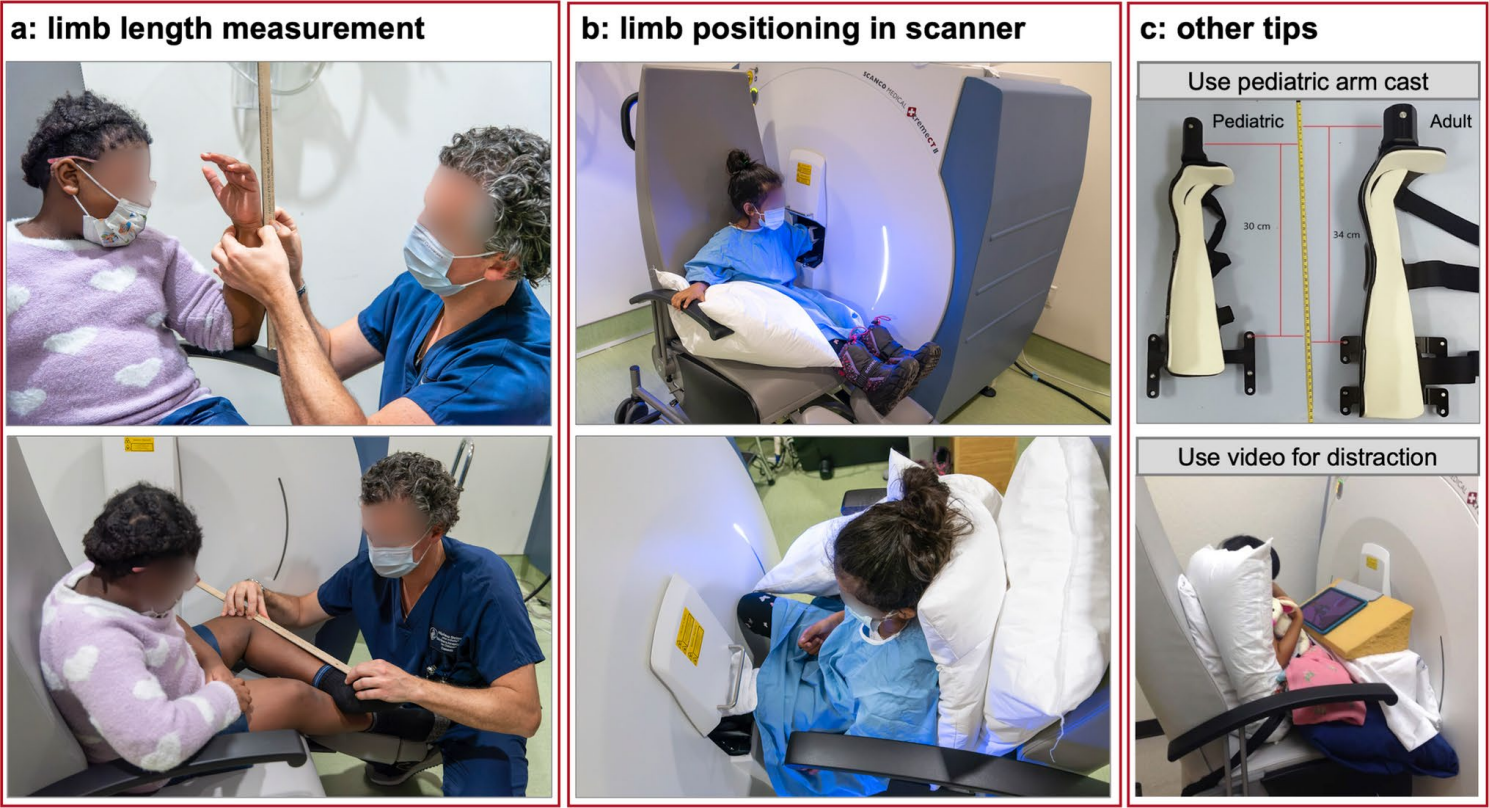
**a** Limb length measurements prior to scanning; **b** positioning of the limb in the HR-pQCT scanner. Pillows can be used to provide support and fill the extra space on the seat to prevent movement; **c** other tips for scanning children to reduce motion include using a smaller pediatric arm cast and playing videos to distract the child

As the growth plate migrates distally during growth, it is rarely possible to measure the exact same bone within an individual over time because the same bone may not exist (e.g., bone located 1 year in the metaphysis may be at the diaphysis the next year and the trabeculae may have been resorbed). In rare cases, image registration can be used to overcome this challenge (see the “Image Registration of Longitudinal Pediatric HR-pQCT Scans” section). However, the general strategy to overcome the limitations of a “moving target” during growth is to scan a percent distance from a bony landmark relative to limb length. This “relative” ROI approach ensures the same relative region of bone is compared between individuals and within the same individual over time. Error is introduced with this approach if the landmark is distal to the epiphysis, as it assumes the distance from the distal end of the bone to the growth plate is proportional to limb length. Bone length cannot be ascertained from the scout view and must be measured manually (Fig. 2a). Limb length measures are highly reproducible (< 1% CV) [30]. Other scanning approaches include landmarking to the most distal or proximal region of the growth plate (or its remnant or scar in older adolescents) and placing the ROI at a fixed or relative region proximal to the growth plate. The primary limitation of this approach is that the shape and position of the epiphysis change over time as does the reference line location as the growth plate fuses. It can be challenging to identify the growth plate, particularly in younger children and for less experienced operators. Thus, there is no clear one-size-fits-all solution to imaging growing children and assessing growing bone longitudinally. The choice of imaging location must be based on the study population and the research objective.

Limb length needs to be measured at each visit. Tibia length is typically measured as the distance between the tibial plateau and medial malleolus by having the participant seated with the ankle of the measurement leg on top of the knee of the other leg (Fig. 2a). The tibial plateau is located by palpating the proximal end of the participant’s tibia. The medial malleolus is identified by palpating the distal end of the medial malleolus. The distance between the tibial plateau and medial malleolus is measured using anthropometric tape or ruler. Although HR-pQCT assesses the radius, anatomical features of the ulna are easier to identify; thus, ulnar length is typically acquired as a surrogate for radial length. The distance between the olecranon process and the styloid process is measured by having the participant place their elbow on a flat surface with their wrist above the elbow and thumb pointing toward the shoulder (Fig. 2a). The olecranon process is in contact with the flat surface, while the styloid process is identified by palpating the distal end of the styloid process (proximal to the 5th metacarpal). The distance between the flat surface and the ulnar styloid process is measured using anthropometric tape or ruler at a 90° angle to the flat surface. In children with limb deformities, we recommend measuring the tibia and ulnar length in the same manner as described above, regardless of the deformity severity.

To acquire HR-pQCT images, the skeletal site of interest (tibia or radius) is first immobilized in a carbon fiber cast and placed inside the scanner’s gantry (Fig. 2b, c). A 2D anterior–posterior scout view scan is performed to identify the ROI. Once the reference line is placed, 110 tissue slices (168 slices in XCTII) are scanned proximally. In total, an approximate 9.02 mm (10.2 mm in XCTII) length of bone of the tibia or radius is scanned in less than 3 min.

The University of California, San Francisco, created reference line training for new HR-pQCT operators to help identify correct bony landmarks on scout views and correctly place reference lines (http://webapps.radiology.ucsf.edu/refline/) [31, 32]. The current module can be used for placing the reference line at the proximal margin of the radial head or tibial plateau (UBC Protocol; Table 1), while future modules will include positioning of the reference line relative to the growth plate (Shriners and Stanford-UCSF; Table 1).

**Table 1:** Description of current distal pediatric protocols (UBC-Calgary, Shriners, Stanford-UCSF)

Protocol	UBC-Calgary	Shriners	Stanford-UCSF
Radius reference line position	Medial proximal margin of radial head	Open GP: Distal margin of GP	Open GP: Proximal margin of GP
		Fused GP (visible): Distal margin of GP	Fused GP (visible): Proximal margin of GP
		Fused GP (not visible): Medial proximal margin of radial head	Fused GP (not visible): Medial proximal margin of radial head
Radius ROI position	XCTI: Ends at 7% of ulna length from the reference line	Begins at 4% ulna length from reference line	Centered at 4% ulna length from reference line
	XCTII: Begins at 4% of ulna length from the reference line		
Advantage(s)	• Same reference line for all ages	• ROI is in the same region with respect to the GP in all participants	• ROI is in the same region with respect to the GP in all participants
	• Reference line placement is more precise	• Excludes GP in all participants	• Excludes GP in all participants
	• Reference line is the same as the standard adult relative offset	• Easy to apply for fully fused or fully open GP	• Fused GP (not visible): Reference line and ROI is the same as the standard adult relative offset
		• Fused GP (not visible): Reference line is the same as the standard adult relative offset	
Disadvantage(s)	• ROI may encroach on the GP in younger (< 8 years) or shorter children	• Reference line changes if GP is not visible at follow-up	• Reference line changes if GP is not visible at follow-up
	• ROI is not in the same region with respect to the GP in all participants	• Reference line placement at the GP is less precise	• Reference line placement at the GP is less precise
	• ROI is not the same as adult scans		
Tibia reference line position	Tibial plateau	Open GP: Distal margin of GP	Open GP: Proximal margin of GP
		Fused GP (visible): Distal margin of GP	Fused GP (visible): Proximal margin of GP
		Fused GP (not visible): Tibial plateau	Fused GP (not visible): Tibial plateau
Tibia ROI position	XCTI: Ends at 8% of tibia length from the reference line	Begins at 4% tibia length from reference line	Centered at 4% tibia length from reference line
	XCTII: Begins at 6% of tibia length from the reference line		
Advantage(s)	• Same reference line for all ages and same as adult tibia scans	• ROI is in the same region with respect to the GP in all participants	• ROI is in the same region with respect to the GP in all participants
	• Reference line placement is more precise	• Excludes GP in all participants	• Easy to apply for fully fused GP (visible/not visible) or fully open GP
		• Easy to apply for fully fused GP (visible/not visible) or fully open GP	
Disadvantage(s)	• ROI may encroach on the GP in younger (< 8 years) or shorter children	• In rare cases the reference line landmark changes between two time points if the GP is not visible	• In rare cases the reference line landmark changes between two time points if the GP is not visible
	• ROI is not in the same region with respect to the GP in all participants	• Reference line placement at the GP is less precise	• Reference line placement at the GP is less precise
Reference data	• XCTI reference data for ages 10 to 21 years [9]	• pQCT radius reference data (XCT-2000) for ages 6 to 23 years [33–35]	• XCTII reference data for ages 5 to 20 years (unpublished)
		• XCTII reference data for healthy children and those with osteogenesis imperfecta from ages 5 to 18 years (unpublished)	
Recommended use case(s)	• Cross-sectional and longitudinal studies spanning late childhood to adulthood	Radius	Radius
		• Cross-sectional: all ages	• Cross-sectional: all ages
		• Longitudinal: studies during growth	• Longitudinal: studies during growth
		Tibia	Tibia
		• Cross-sectional and longitudinal studies of all ages	• Cross-sectional and longitudinal studies of all ages

*UBC*, University of British Columbia; *UCSF*, University of California—San Francisco; *ROI*, region of interest; *GP*, Growth plate; *XCTI*, first-generation HR-pQCT; *XCTII*, second-generation HR-pQCT; *pQCT*, peripheral quantitative computed tomography

## Current Pediatric HR-pQCT Protocols

For all pediatric protocols, the non-dominant tibia and radius are typically scanned unless the participant sustained a previous fracture of the tibia or radius or has a metallic implant in the ROI, in which case the opposite limb is scanned. The dominant tibia is typically identified as the preferred leg for kicking (i.e., “which leg would you use to kick a soccer ball”).

### University of British Columbia Protocol

**Fig. 3:**
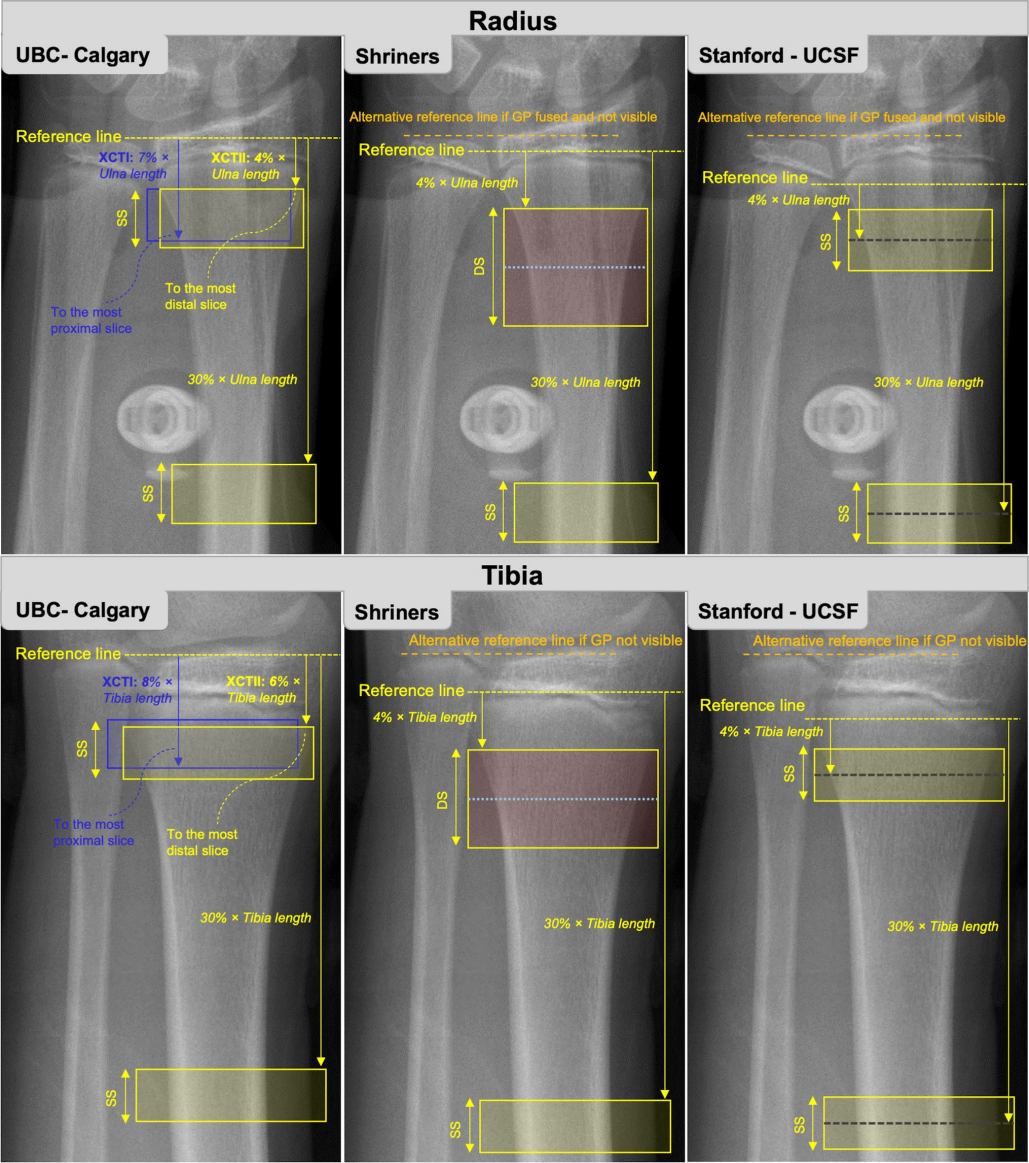
Reference line placement for current pediatric HR-pQCT protocols at the radius (top row) and tibia (bottom row). UBC, University of British Columbia; XCTI, first-generation HR-pQCT; XCTII, second-generation HR-pQCT; GP, growth plate; SS, single stack; DS, double stack

The University of British Columbia (UBC) developed a protocol using a relative ROI for children and adolescents using XCTI. In this protocol, the reference line location is placed at the distal tibia plateau or medial proximal margin of the distal radius (Fig. 3). Scans proceed proximally toward the 8% site of the distal tibia (% of total tibia length) or 7% site of the distal radius (% of total ulnar length), such that the 8% and 7% sites are the most proximal scan slice. The 8% and 7% ROI include both cortical and trabecular bones and exclude the growth plate in most children [28, 30]. This protocol was used throughout the mixed-longitudinal UBC Healthy Bones Study [1, 3, 36–42], for developing normative data for XCTI in children and adolescents [9], and by other research groups [43–46].

To avoid encroaching on the growth plate with an additional 1.0-mm scan region for XCTII, the original UBC protocol has been adapted for XCTII by adding the additional 1.0-mm scan region to the proximal end of the scan. Further, there was some confusion around the location of the ROI in the original XCTI protocol (e.g., the last slice of the scan was the 8% or 7% site as opposed to the first slice); thus, the protocol for XCTII has been adjusted to begin at the 6% site of the distal tibia and 4% site of distal radius and scan proximally from there. With this revised approach, the most proximal slice of the scans ends near the 8% and 7% sites of the distal tibia and radius, respectively, as in the XCTI protocol (Fig. 3). Although microarchitectural parameters differ between XCTI and XCTII, total BMD should remain comparable between the two scanners and can be related to existing XCTI normative data (spanning age 9 through 21 years) [9].

### Shriners Protocol

The Shriners Hospital for Children-Canada (McGill University) developed another variation of relative ROI positioning for children using XCTII. In this protocol, the reference line location depends on the status of the growth plate. If the growth plate is open, the reference line is placed at the most distal margin of the distal growth plate for both the radius and tibia (Fig. 3). When the growth plate fuses with no visible remnant, it can be difficult to identify the most distal margin; thus, the reference line is placed at the medial proximal margin of the radial articular surface and at the tibial plateau, which are locations recommended for reference line placement in adults (Fig. 3) [8•, 31]. The scanned region begins at 4% of ulna or tibia length from the reference line and proceeds with a double stack (two sequential scans; 20.4 mm stack length; see the “Double Stack Scanning” section). Note that a single stack can be used instead of a double stack, since we encourage reporting each stack separately. Similar protocols for the radius and tibia make the protocol more straightforward for radiology technicians to perform. Also, extensive reference data from pQCT are available for the 4% region in children [19, 33–35, 47]. This protocol excludes inadvertently scanning the growth plate in all participants. One disadvantage of the protocol is that if the growth plate fuses and becomes indistinguishable during the study period, one must switch landmarks for reference line placement (from the pediatric to adult protocol) for a participant’s subsequent imaging. However, at the radius, the distance between the most distal margin of the growth plate and the medial proximal margin of the radial endplate is small; thus, switching between landmarks when the growth plate fuses results in minimal change to the volume of interest scanned. In practice, the need for switching between landmarks is rarely required since even in case of growth-plate fusion, some remnants will be visible such that using the pediatric landmarks is feasible.

### Stanford/University of California San Francisco Protocol

The Stanford SAMBA Lab and University of California San Francisco (UCSF) developed another variation of relative ROI positioning for children and adolescents that uses the proximal margin of the growth plate as the reference line. The reference line is set at the most proximal edge of the growth plate, growth plate remnant, or scar (Fig. 3). The scanned region is centered at 4% of ulna or tibial length from the reference line. This location centers the scan ROI in a standard location in the metaphysis with respect to the endochondral ossification front. The proximal edge of the growth plate can be challenging to locate when the growth plate is fusing. Further, the most proximal edge may be medial, central, or lateral, so technicians must be trained to look at the entire growth plate. If the growth plate is fused and there is no visible growth plate, growth plate remnant, or scar, then the reference line would move to the endplate (adult reference line) and the standard adult relative offset protocol would be used. This occurs more often at the radius and almost never at the tibia. A reference line placement module will soon be available to help train operators locating this region.

### Diaphyseal Scan Site

With the longer XCTII gantry, cortical bone parameters can be assessed at diaphyseal sites. The UBC and Shriners protocols assess diaphyseal bone at 30% of ulna or tibia length relative to their respective reference lines, while the Stanford protocol centers the stack at 30% of ulna or tibia length relative to the reference line. Outcomes of interest at the 30% site include total and cortical area, cortical BMD, thickness, and porosity.

### Motion Artifacts

Any participant movement during high-resolution imaging increases the likelihood of motion artifacts (streaks or discontinuities on the scan), which may require that scans be repeated or excluded from analysis. A second scan is typically acquired if there are significant motion artifacts (≥ grade 3 on a 5-point grading scale with a score of 5 the poorest quality; see Pauchard et al. and Sode et al. for illustration) [48, 49]. Our sites acquire up to a maximum of one or two re-scans. Scans with a motion artifact of grades 4 or 5 are usually excluded. The radius is more susceptible to motion artifact than that tibia and scan quality improves throughout childhood and adolescence.

## Image Processing and Analysis

In the original XCTI standard analysis, the manufacturer’s (Scanco Medical) protocol separates cortical from trabecular bone using a semi-automated threshold-based algorithm equivalent to 1/3 the apparent density of cortical bone [50]. This step requires hand-drawn contours of the periosteal surface of the bone. The following parameters are directly measured from the standard analysis: total BMD (Tt.BMD; mgHA/cm^3^), trabecular BMD (Tb.BMD; mgHA/cm^3^), and trabecular number (Tb.N; 1/mm). Tb.N, the mean number of trabeculae per mm, is a truly 3D measure and is calculated as the inverse of the mean spacing between the mid-axes of trabeculae. Trabecular bone volume fraction (Tb.BV/TV; %), trabecular thickness (Tb.Th; mm), and trabecular separation (Tb.Sp; mm) are derived variables. Tb.BV/TV is estimated as the ratio of Tb.BMD (mgHA/cm^3^) to 1200 mgHA/cm^3^. It is not possible to directly measure Tb.Th using XCTI because the HR-pQCT voxel size approximates the average thickness of individual trabeculae; therefore, Tb.Th is derived using the histomorphometric plate model [12]. In contrast to XCTI, Tb.BV/TV (%) is calculated directly for XCTII as the ratio of voxels in the mineralized bone phase to the total number of voxels in the trabecular region. Tb.Th (mm) and Tb.Sp (mm) are also calculated directly using voxel-based measurements using the distance transformation method [11, 51]. Automatic segmentation of the periosteal contour is also available for XCTII, although visual inspection of the produced contours by the user is required. Both XCTI (alternate segmentation) and XCTII (default segmentation) use a dual threshold algorithm or “extended cortical analysis” to separate the cortical and trabecular compartments [14]. However, technicians must visually inspect all contours for errors and manually correct where needed. For example, cortical bone may be included in the trabecular mask in regions where the cortex is thin. Importantly, new HR-pQCT users should be aware that the XCTII system may not have all required scripts installed for re-creating cortical and trabecular masks once contours are corrected, but rather they may need to ask the manufacturer for these scripts. Outcomes from the extended cortical analysis include total area (Tt.Ar; mm^2^), cortical area (Ct.Ar; mm^2^), cortical porosity (Ct.Po, as the number of void voxels within the cortex; %), cortical BMD (Ct.BMD, apparent density of the cortex including all pore space; mg HA/cm^3^), and cortical thickness (Ct.Th, directly measured after removing intracortical pores; mm). Ct.Th and Ct.Po are highly correlated with micro-CT parameters in adult bone cadavers (*r* = 0.80 and *r* = 0.98, respectively) [52]. Density parameters and some microarchitectural measures can be compared and/or converted between XCTI and XCTII [53]; however, resolution-dependent outcomes such as Tb.Th cannot be compared between systems. Validity of trabecular and cortical bone parameters in the growing skeleton is currently unknown. Further, the default adult XCTII segmentation approach, based on a BMD-threshold, might not be suitable during growth when tissue density and partial volume effects may vary with age.

Image filtration and binarization also differ between XCTI and XCTII. For XCTI segmentation, Laplace-Hamming filtering (Laplace *ε* 0.5, Hamming cutoff 0.4) is used for noise reduction and edge enhancement prior to the application of a global threshold, whereas XCTII images are filtered for noise reduction using a low-pass Gaussian filter (sigma 0.8, support 1.0) and fixed different thresholds to extract trabecular and cortical bone (320 and 450 mgHA/cm^3^, respectively). Advantages of the XCTII approach include shorter processing time and simpler thresholding of images based on bone density values. However, a recent study demonstrated that Laplace-Hamming filtering of XCTII scans substantially improves segmented images, including more accurate segmentation of fine trabecular (BV/TV, Tb.Th) and cortical (Ct.Po) features [54•]. Given that trabeculae are thinner in children than adults [24], Laplace-Hamming segmentation of XCTII scans might improve segmentation and future studies should investigate Laplace-Hamming segmentation methods in children.

## Finite Element Analysis

Conceptually, finite element (FE) analysis breaks down a complex structure (i.e., bone) into smaller simpler elements and estimates the distribution of forces and displacement throughout the complex structure while accounting for morphology, material properties, and loading conditions. Material properties and loading conditions are typically defined by the user. FE analyses applied to HR-pQCT images can directly estimate bone strength (i.e., failure load), which is a stronger predictor of fracture risk than density and morphological measurements in adults [55, 56]. The ratio of reaction force and applied displacement is known as stiffness (kN/mm). Apparent modulus [MPa] is also often reported, which is the average stress divided by the applied strain. Ultimate stress [MPa] has been reported in several pediatric studies [1, 3, 41–43] and is related to the apparent modulus via an empirically derived equation against experimentally determined bone strength [15]. Lastly, the ratio of force carried by each bone compartment (i.e., trabecular or cortical) as well as specific sub-regions (i.e., distal or proximal) can be estimated.

Micro-finite-element (μFE) models require minimal preprocessing, as the models are voxel based, meaning the geometry is defined by directly converting the isotropic voxels of segmented HR-pQCT images into the same size cubic hexahedral elements (also known as brick elements). The number of elements is typically in the range of 1–10 million, making the models very large for available commercial FE solvers. Accordingly, specialized solvers have been developed to improve processing time [57]. HR-pQCT-based μFE models typically simulate compression of bone along the longitudinal axis of the scanned bone region. The application of such boundary conditions is straightforward and can be done automatically. With μFE axial conditions, any lateral displacement is suppressed at the top and bottom surfaces to simulate high-friction setup. While one end is fixed along the longitudinal axis, a constant displacement of 1% of the total height of the model is applied to the other end; hence, 1% compressive strain. The only difference with the uniaxial μFE approach is that the top and bottom surfaces can undergo lateral displacement to simulate low-friction setup. Outcomes from both models are highly correlated (*R*^2^ > 0.99) [58].

Accurate material properties including Poisson’s ratio and elastic modulus (also known as Young’s modulus) are required for μFE models. Inputs are usually identified by validating against experimentally determined measures of bone strength or local strains from adult cadaveric bone. Acquiring pediatric cadaveric specimens is extremely difficult; thus, pediatric studies reporting μFE-based parameters currently apply the same material properties and failure criterion as adult studies (e.g., Poisson’s ratio of 0.3 and elastic modulus of 10,000 MPa) [1, 3, 9, 39, 41–43, 59–63]. Adult material properties are assumed to be homogeneous and constant; this assumption may not be true during growth, as tissue density is variable and changes with age. Future studies should investigate material properties of pediatric bone, including examining how rare diseases such as osteogenesis imperfecta affect material properties and subsequent μFE-based bone strength estimates [64, 65].

Although linear μFE models are most common, nonlinear μFE models include bone material post-yield behavior (i.e., the material stress–strain curve will not be linear). Nonlinear models are more complex and computationally demanding but show modest improvements in bone strength prediction [15]. Linear μFE models estimate bone strength using empirical relationships between FE-derived bone stress or strain and some strength metric (e.g., bone yield stress or strain). Failure load is estimated by multiplying a “failure factor” by the total reaction force from 1% strain. Failure is assumed when a predefined volume of bone tissue (critical volume) exceeds a specified critical strain. This failure criterion is known as the “Pistoia criterion,” and the most widely used values are a critical volume of 2% and a critical strain of 0.7% [62]. The failure factor is computed by dividing the critical strain by the actual strain at critical volume. Again, it is unknown whether the failure criterion used to estimate bone strength in adults is accurate for pediatric bone, and future studies should investigate pediatric bone-specific failure criterion. An alternate HR-pQCT-based FE model is homogenized FE (hFE) [66, 67]. hFE homogenizes the bone volume fraction and trabecular orientation inside a predefined volume of elements several times larger than the original voxel size to map these properties to the continuum elements at the macroscopic level. hFE models are promising because they require less computational resources than μFE models and can be used to develop nonlinear material models.

## Image Registration of Longitudinal Pediatric HR-pQCT Scans

**Fig. 4:**
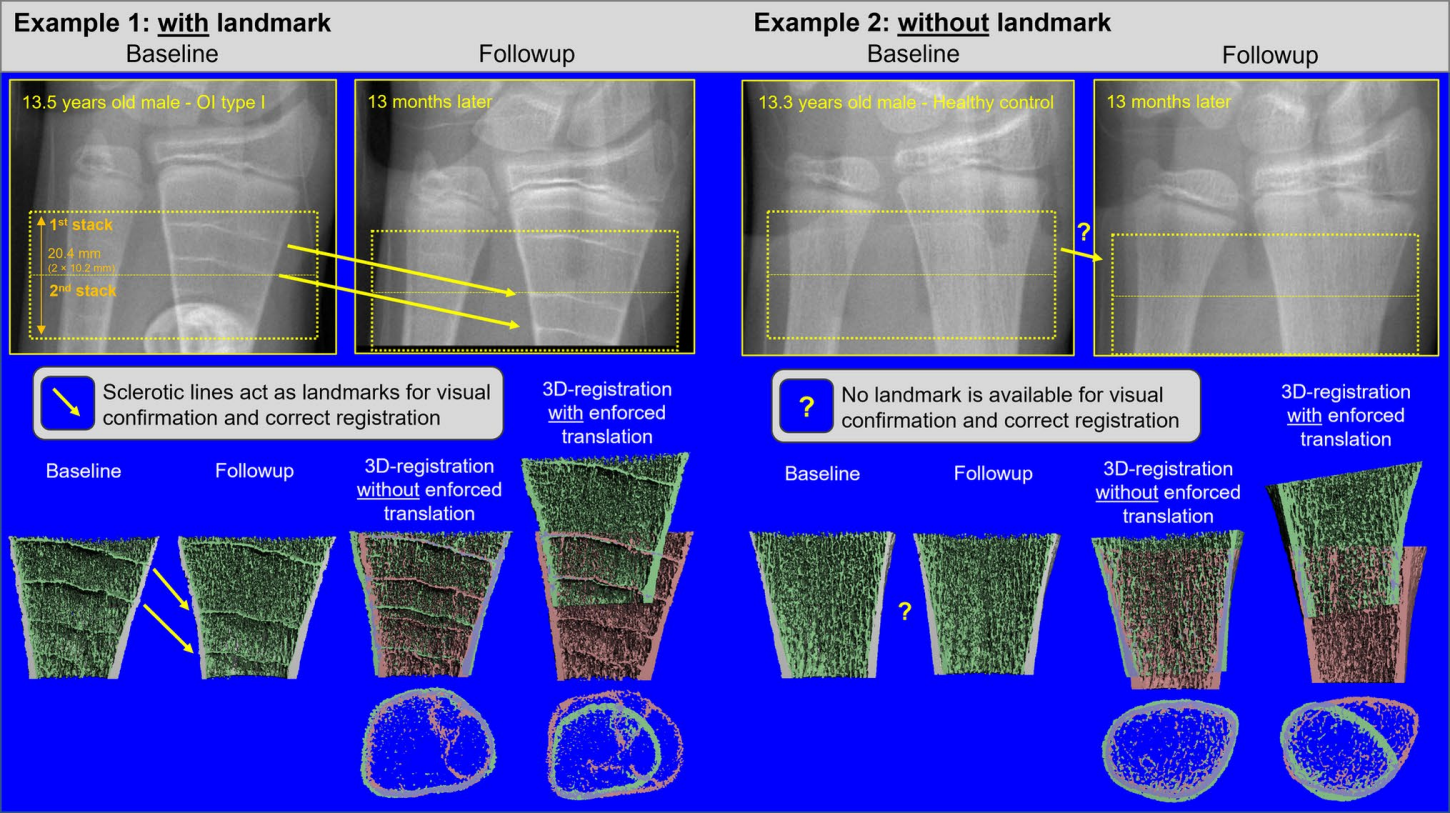
Examples of 3D image registration at the distal radius with and without landmarks. In the example with landmark, a 13.5-year-old boy with type I osteogenesis imperfecta (OI) is scanned at two time points, 12 months apart. Due to bisphosphonate treatment, the remnants of the growth plate are visible on the scans as distinct lines. These lines provided enough features between the scans for correct image registration, when accompanied by proper initial translation enforced by the user. The line could also be used for visual verification of the registration. For the case without landmark, a 13.3-year-old healthy boy was scanned at two time points, 13 months apart. In this case, 3D registration failed to properly align the scans. Due to the lack of any distinct landmark, proper registration could not be visually verified

In adults, inaccuracies arise in successive HR-pQCT scans in longitudinal studies due to changes in limb positioning (shifts along the longitudinal axis or rotational misalignment) that make it challenging to image the exact same volume of bone over time [68]. Two- or three-dimensional (2D/3D) image-registration approaches are applied to longitudinal bone images to align two images to the same image space based on similar features or intensities [68, 69]. Due to growth and bone (re)modeling as mentioned in the above section (“Image Acquisition”), it is not recommended to use current image-registration approaches (2D, cross-sectional area (CSA) slice matching or 3D translation and rotation) in growing children. The CSA registration method cannot be applied to a growing bone cross-section as it assumes a constant bone area [70]; thus, users should select the “no match” analysis option for longitudinal scans. 3D registration is also difficult to successfully apply during growth since the bone area cannot be used to match two scans and there are no common landmarks between subsequent scans. Additionally, the more commonly used “rigid” registration is not optimal for registering growing bone because morphing is a non-rigid change. Nevertheless, identifying the same bone volume might be of interest in certain circumstances. In a preliminary study at Shriners Hospital for Children-Canada, we investigated the feasibility of rigid 3D registration using Scanco image processing language (IPL) on images taken at baseline and 1-year follow-up in children with osteogenesis imperfecta (OI) and age- and sex-matched healthy controls. In healthy children, the rigid 3D registration failed to properly align the scans from two time points. In children with OI, the rigid registration was only successful if specific landmarks could be matched between two time points, along with assignment of initial translation by the user (Fig. 4). In such cases, the landmarks were sclerotic lines, which are horizontal trabeculae containing some degree of cartilage, thought to be caused by the temporary interruption of growth plate cartilage resorption during bisphosphonate treatment [71]. Thus, there may be scenarios when landmarks exist and using 3D image registration is appropriate. Only in these rare cases can more advanced methods such as timelapse HR-pQCT-based morphometric analysis be used since it requires alignment of scans from two time points. Of note, while sclerotic lines assist image registration, they can lead to overestimation of BMD in children with OI. Finally, without available techniques to align successive scans during growth, measurement precision in longitudinal investigations undoubtedly suffers and is an area of future research need.

## Pediatric HR-pQCT Precision Data

Two studies reported XCTI HR-pQCT short-term precision for children; both studies used the same repeated scans from 8- to 14-year-old healthy boys and girls [43, 45]. Precision errors in children and adolescents were similar to unregistered precision errors in adults [53], with precision for density at the radius and tibia ranging 0.6 to 1.8%, area 1.5 to 5.8%, microarchitecture 2.3–9.1%, and μFE failure load 2.6–2.7% [43, 45].

For XCTII, precision errors from unregistered repeated scans are similar to those previously reported for XCTI [72]. Improved precision was observed at the 30% sites (< 1% for density, cortical thickness, and failure load, and < 6% for cortical porosity) compared to distal sites due to more homogenous bone composition at shaft sites (and no trabecular bone) [72]. Precision data do not currently exist for children with rare bone diseases.

## Special Cases

### Double Stack Scanning

The traditional HR-pQCT scanning region is a single-image stack, covering a length of 9.02 mm or 10.2 mm of bone for XCTI and XCTII scanners, respectively. Due to the relatively low radiation dose of HR-pQCT, a larger bone length can be scanned with two image stacks and can be useful when scanning children over time (double-stack scanning is standard procedure in the Shriners protocol), as the ~ 1 cm/year growth in bone length is similar to the height of a single stack. A double stack requires at least two full rotations of the gantry and thus motion artifact can cause slight misalignment of the two consecutive image stacks. The two stacks must be reported separately for FE outcomes because of possible misalignment between the stacks. However, other outcomes can be reported separately per stack or combined, allowing for inter-study comparability if only a single distal stack is desired.

### Rare Bone Disease Populations: Osteogenesis Imperfecta

When imaging children with rare bone diseases, additional care is often required to safely scan the patients and accurately measure bone outcomes. An example is children with osteogenesis imperfecta (OI), which is a collagen-related genetic disorder resulting in bone fragility. As with most rare bone diseases, HR-pQCT data for children with OI are sparse. While numerous studies using pQCT have been performed in children with OI, only one cross-sectional study of 9 children with mild (*N* = 7) and severe (*N* = 2) OI, aged between 9 and 15 years reported outcomes using HR-pQCT (XCTI) [73•]. Fennimore et al. demonstrated feasibility of positioning and scanning and did not report any difficulty scanning children with OI [73•]. However, since children with OI have higher fracture incidence, their non-dominant limb was often not scanned because of a recent fracture or presence of a metal implant. These findings concur with our observations scanning children with OI using pQCT and HR-pQCT at Shriners Hospital for Children-Canada.

## Practical Tips for Pediatric Scanning

Reducing participant motion is one of the main challenges acquiring usable HR-pQCT scans in children. It is essential to understand the mental and physical characteristics of the pediatric population to be scanned and adjust preparation and communication accordingly. Several strategies we have found useful include the following:

- Sending the child and caregiver pictures of the scanner with a child in it in advance of their visit. Some are fearful of the scanner itself and images can help both children and their caregivers prepare for what to expect.
- Making the scanning room child friendly (e.g., stickers on the wall or device).
- Providing distractions such as a video that participants can watch without touching or moving their upper body (e.g., propped on a foam block or a tablet stand; Fig. 2c).
- Describing what the machine will do (e.g., “this machine will take a picture of the bones in your leg. The scanner makes a funny sound, but nothing will touch you during the scan.”)
- Identifying any wiggly toes or thumbs that can be controlled with some tape.
- Making the child as comfortable as possible, particularly for the radius scan. Pillows can be tucked around their arm or leg to help support the limb. A box to rest the feet can help prevent the legs from swinging. Blankets might be needed as the room is often cool.
- Using the smaller arm cast for the radius scan.
- If using the manufacturer’s chair, placing pillows to prop the child closer to the gantry (Fig. 2b, c).
- For participants with mobility challenges, preparing ahead for the use of alternate seating (e.g., wheelchairs, EOS chair) or transferring to the manufacturer’s chair and stabilizing the participant with Velcro straps or a harness.
- If the first scan has a motion score of 3 or higher, checking in with the participant to see if they are cold, fearful, or have any questions. Ask if anything needs to be changed and try again. More than anything, having patience and kindness working with these participants to help them remain still.

### Feasibility

Our data at Shriners suggest that for scanning individuals with an ulna length of less than 18 cm, the feasibility depends on their flexibility. If flexible, upper limbs as short as 13 cm may be scanned by (1) positioning the arm lower in the cast and (2) altering the position of the frame of the small arm cast. Similarly, for individuals with a tibia length less than 24 cm, the feasibility of scanning will depend on their flexibility and comfort with the cast reaching their upper thigh.

## Summary and Future Directions

HR-pQCT is a valuable tool to improve our understanding of how bone is accrued during childhood and adolescence. Imaging growing bone is complicated, and no single pediatric imaging protocol fits all scenarios and research questions. However, in this review we provide three protocols that have been used successfully in pediatric populations. We recommend that new investigators consider using one of the existing protocols to facilitate inter-study comparisons rather than developing a new protocol. Regardless of the imaging protocol, longitudinal data should be reported without registration using the “no-match” option. Future multicenter studies using HR-pQCT in children will need to consider uniformity of methods and data cross-calibration between scanning centers. Current cross-calibration strategies used in adult studies [74•] would be improved by using cadaveric bone phantoms that have a range of bone densities including those representing pediatric bone. Finally, as use of HR-pQCT imaging within pediatric populations grows, researchers should consider investigating the following areas of need:

- Studies in pediatric populations with common and rare diseases.
- Precision data in healthy children and in children with common and rare diseases.
- Studies assessing the validity of image segmentation, including applying Laplace-Hamming or new BMD-independent segmentation methods with XCTII scans.
- Studies assessing the validity of adult homogenous material property assumptions and μFE failure criterion in children.
